# A Retrospective View of the Late Discoveries and Improvements in Chemical Philosophy

**Published:** 1818-08

**Authors:** W. Hutchinson

**Affiliations:** Berners'-street.


THE LONDON

### Medical and Physical Journal.

2 OF VOL. XL.] AUGUST, 1818. [NO. 234.

"For many fortunate discoveries in medicine, and for the detection of numerous errors, the world is indebted to the rapid circulation of Monthly Journals; and there never existed any work to which the Faculty in Europe and America were under deeper obligations than to the Medical and Physical Journal of London, now forming a long, but an invaluable, series."—Rush.

*For the London Medical and Physical Journal.*

### *A Retrospective View of the late Discoveries and Improvements in Chemical Philosophy;* by W. Hutchinson, Esq., of Berners'-street.

"Multum adhuc restat operæ, neque ulli nato post mille secula præcidetur occasio aliquid adhuc adjiciendi."

On taking a general view of the rise and progress of science and the liberal arts, we cannot fail being induced to dwell with admiration and delight on those periods in civilised nations, at which, from a certain state of mental cultivation, and the emulation excited by the acquirements of some pre-eminent genius, the power of the human intellect, in dispelling the shades in which Nature has veiled her operations, has been so effectually displayed. The æras of Alexander in Greece, of Augustus Cæsar in Rome, of the Medici family in Italy, and Newton in England, will ever be a cause of exultation for the advocates of civilized life, in opposition to those sentimental theorists, who extol barbarism under the name of simplicity, and undervalue the advantages of a refined and cultivated age.

Commensurate to the pleasure we experience in the contemplation of such a view of human knowledge is our regret on considering that these have been eminences beyond which our guides have in vain attempted to advance, and from which subsequent votaries have wandered into the paths of error and obscurity. This, as regards many subjects for inquiry, may most probably be attributed to the confined nature of the human powers.—All that the mind of man is capable of comprehending respecting most of the pure sciences, was understood by the Greeks: the Italians, during the period to which I have alluded, carried the execution of the liberal mechanic arts to a degree of refinement that can never be excelled. The mathematics, the ground-work of science, and once the study of all who aspired to true knowledge, are now but languidly cultivated. We have not the cheering hope to soothe our labour, of being able to make any discovery that we may show to the world as the fruit of our toil. They were brought to perfection by Newton, and have since been gradually falling into decay.

Such being the fate of most of the objects of our study, how gratifying it is to reflect that there exists one branch of philosophy built on a basis that time cannot overthrow, and embracing subjects for investigation that human industry cannot exhaust for ages to come: still, as we advance,

> " New distant scenes of science strike our eyes,
> Hills peep o'er hills, and Alps on Alps arise."

Such is PHILOSOPHICAL CHEMISTRY; and we may, without presumption, assert that the present period will hereafter be considered an important æra in the history of science. This is a source of gratification for its votaries, which, with the interest attending the application of this science, and the light it reflects on the operations of nature in general, has made it the study of all men of liberal education. Could BACON have lived to see the consequences which have ensued from following his precepts, he would have acknowledged that his labours in opening a path to the regions of science, through the wrecks that time, ignorance, and superstition had thrown in the way, were amply repaid.

It would indicate vanity and presumption were I to attempt to describe the absolute merit and importance of this branch of science, after what has been said respecting it by so many enlightened philosophers and eloquent writers: I would merely endeavour to point out its relative excellence, and to show that neither genius nor industry will study it, without making acquirements and discoveries that will amply repay their toil: that saddening thought, that

> " ——— Οὔτ' ἐπιδερκτὰ τὰ δ' ἀνδράσιν, οὔτ' ἐπακουστὰ,
> Οὔτε νόῳ περιληπτά·"

cannot be felt by the students of chemistry.

The disposition of philosophers to seek for a single and distinct principle of action or agency in natural operations, —an axiom, in the greater number of instances, most probably founded in truth,—has in all ages been carried too far in the study of animal physiology. The human body, doubtless the most complex combination of matter in nature, adapted to such a variety of functions, each of which is effected by distinct organs, but between which there is a general relative connexion or sympathy of action, can hardly be supposed to have all its actions performed by any one general principle. All the systems of physiology that have been raised from such a foundation have fallen into ruins. A few years since, the cause of every natural and morbid animal action was sought for in chemical operations: the nature of the different parts of the body, and its natural and morbid secretions, were examined with the most scrupulous attention; and an attempt was made to raise a system of therapeutics on this basis. The consequence of this was, an ardour for the study of chemistry amongst professors of medicine that had never been before observed. But the fallacy of this system has been generally acknowledged, and the study of that science by medical enquirers, in general, has fallen to that which had previously been its object—the direction of pharmacy. But what has had the greatest influence in diverting medical practitioners from it, is the rapid progress it has made within the last few years; to keep up with which would require more regular and constant attention than men in general, engaged in the practice of medicine, can devote to it. Besides this, the atomic theory, which may be called its guide, not being generally understood, has thrown a shade over its operations, which conceals them from the view of common observers.

A conviction of the real importance of a knowledge of the principles of chemistry in the practice of medicine, has induced me to use my feeble endeavours to call the attention of its professors to this subject; and, to those who are acquainted with its progress until a late period, I have considered that I should render an acceptable service, in collecting into one channel the streams that spring from a variety of sources, devoid certainly of their sparkling lustre and golden sands, but capable, when well directed, of fertilizing the regions through which they may flow.

My readers, I presume, are acquainted with the powerful agency of the *blow-pipe* in chemical operations, when, in place of air, a mixture of oxygen and hydrogen gases is employed. This discovery, the promulgation of which is due to Dr. Clarke, will in many instances supersede the more elaborate application of electricity. Various modes have been suggested for a construction of it which would prevent the hazard of explosion of the mixed gases: the most simple and efficacious appears to be that of Dr. Watt, constructed on the principle of the safety-lamp, a particular account of which appeared in the 232d Number of your Journal.

*Metallic Thermometer.*—A very beautiful and delicate instrument of this kind has been constructed by M. Breguet,

It is made with slips of two metals twisted into a spiral form, at the extremity of which is fixed an index, which moves round a graduated circle as the spiral is more or less twisted or untwisted by the influence of different degrees of heat. The metals used are silver and platinum, and, to render the extreme points more fixed, and prevent sudden motions of them, a slip of gold, the expansibility of which is intermediate between that of silver and platinum, is soldered between these two metals. This is more delicate than either the mercurial or air thermometers. It was placed with a mercurial one under the receiver of an air-pump, when the temperature of the atmosphere was 66.2°. On exhausting the receiver, the mercurial thermometer fell 3.6°, but the spiral one 41.4° of Fahrenheit.*

In experiments with the *gases*, much time and care are required to ascertain their mean bulk, and the variations resulting from changes in the temperature and pressure of the atmosphere; and, in delicate experiments, it is frequently desirable to know whether any slight change in the volume of the gas be owing exclusively to the agency of an external cause, or whether there be an absorption or evolution of gas, by the materials subject to the experiment. An instrument has been invented by Dr. Marshall Hall, by the aid of which the necessity for distinct calculations may be superseded. It would be difficult to render the application of this instrument intelligible to general readers, without a graphical representation of it; those who feel interested in it, therefore, are referred to the work† in which the account of it was communicated to the public.

A knowledge of the phenomena attending the development of the form of regularly crystallized bodies, and the laws by which they are regulated, constitutes a very interesting and important part of the study of chemical science. Dr. Ure has published an account of some experiments he instituted with a view to ascertain those laws, as regards the crystallization of saline substances, the results of which promise to lead to some important discoveries.‡

It is well known, that if a hot saturated solution of some of the neutral salts be put in a phial, and well corked, it may be cooled many degrees below the crystallizing temperature without losing its fluidity; but if the cork be then withdrawn, crystallization immediately takes place; and, at the instant of solidification, the temperature rises from 30 to 40

* *Ann. de Chimie et Phys.* v. 312.

† Journal of Science and the Arts, No. 9.

‡ Ibid.

degrees; affording, as generally considered, a fine illustration of the doctrine of latent heat. This phenomenon has been ascribed to the sudden abstraction of a portion of heat from the liquid on the admission of external air, whence the crystallization is determined at the surface, and proceeds gradually downward. The experiments of Dr. Ure prove that it arises from other causes. A phial of a saturated solution of sulphate of soda, covered with bladder, was placed under the receiver of an air-pump; on piercing the bladder no change took place, but, on agitating the vessel, a portion of the solution was thrown out with an explosive effort upon the receiver; the liquid instantly shot into a confused state of crystallization, and at the same time began to boil from the heat evolved during the process. In another experiment the bladder was punctured *in vacuo*, and the air slowly admitted into the receiver without effect; but, on slightly shaking the vial, the liquid passed into the solid state. A solution was put into a phial, which was partially closed with a perforated cork, in which a glass tube, 1-20th of an inch in diameter, and four inches in length, was inserted. After standing fourteen hours thus exposed to the air, its liquidity was unimpaired, and the cork was withdrawn without any change taking place; when finally, on agitation of the fluid, it solidified. A similar solution was covered with olive oil and cooled without crystallizing; but on smartly shaking it this took place: the phial had previously been placed on a vibrating glass without effect.

A glass tube was filled with a similar solution; through a cork at each end of which a platina wire was inserted, and the ends exposed to the opposite electrical influence of a voltaic battery; hydrogen and oxygen gases were evolved from the oppositely electrified wires more abundantly than from pure water with the same voltaic power; after a few seconds, crystallization appeared at the negative end of the vessel, and proceeded slowly and steadily towards the positive end, the place of demarcation between the congealing and liquid part being smooth and vertical. Different experiments were instituted with the same general results. From the consideration of the above phenomena, it would appear probable, that electricity may be found a useful agent in promoting saline crystallization, and may perhaps be employed by nature in her crystalline formations. The effect of mechanical disturbance, in determining saline crystallization, is illustrated by the symmetrical disposition of particles of dust and iron by electricity and magnetism. Strew these upon a plane, and present magnetic and electric forces at a certain distance from it, no effect will be produced. Communicate to the plane a vibrating movement; the particles, at the instant of being liberated from the friction of the surface, will arrange themselves according to the laws of their respective magnetic or electric attractions. The water of solution, in counteracting solidity, not only removes the particles to distances beyond the sphere of mutual attraction, but probably also inverts their attracting poles. Hence, when they are again brought within the attracting limit, by abstracting the water, or the repulsive caloric, some additional force is necessary to revert this liquid arrangement of the poles. It is thus that a crystal brought into contact with the surface of the solution may be supposed to act.

An artificial *classification of mineral substances*, either from their chemical composition or external characters, if of no further importance, serves at least to render the communication of knowledge respecting them more familiar, and to lessen the labour of original investigation. M. Beudant has published an interesting memoir *on the mode of determining the species of mineral substances.** The principles of the classification of those bodies have excited disputes among mineralogists and chemists for upwards of thirty years past: some arguing for the propriety of its being dependant on chemical analysis, others on their crystalline form. The first regular classification of minerals, on the principle of chemical analysis, was published by Bergman in 1782. To this various others have succeeded; but that which Berzelius has recently proposed, in which he considers the oxides of aluminum and silicium as performing the office of acids in the composition of mineral bodies, is unquestionably the most ingenious and perfect that has been founded on chemical analysis. On the principles of this mode, minerals of the same species are defined *to consist of such substances as are similarly compounded*; but it is evident, from the irregularity of the proportions of their constituent parts, that this definition becomes uncertain in its application, and that the determination of a species will frequently be arbitrary. About the same time that Bergman was employed in constructing his mode of classification, Romé de Lisle, in comparing together variously formed crystals of the same substances, discovered that they might all be referred to the same fundamental form, of which they might be considered a modification, resulting from the truncation of its edges or angles; but he did not pursue this idea further, so as to apply it to the definition of a species. This was done by Haüy, who, by a method equally ingenious and exact, de-

* *Annales des Mines.*

monstrated that all the crystalline forms were related to one single system of geometry; that the system varied in relation to different substances, but was uniform in its application to all the varieties of the same species; and he thence concluded that crystallization was the character on which the greatest reliance could be placed in determining the species of a mineral. The definition of a species according to this classification must be *a collection of substances, of which the integrant molecules are similarly composed of similar elements, and in similar proportions.* But even in this mode it is evident that the results both of chemical analysis and crystalline form are regarded, only that the greatest importance is attached to the latter. With the view of illustrating this problem, M. Beudant instituted a series of experiments on such substances as might be compounded and decompounded at pleasure; as these would enable him to determine the proportion of accidental mixture capable of entering into the composition of a body without affecting its crystalline form. He found that, when two different salts were mixed together in certain proportions, and the solutions were crystallized, the crystals obtained never contained the two salts in the same proportions that had been originally mixed, but in some other proportion. He found that crystals might be obtained composed of 85 parts of sulphate of zinc and 15 of sulphate of iron; of 91 parts of sulphate of copper and 9 of sulphate of iron; and of 72 parts of sulphate of copper, 24 of sulphate of zinc, and 3 of sulphate of iron; and yet all of these crystals will have the form of the crystals which distinguishes sulphate of iron. The general results of the experiments of M. Bendant led him to prefer the classification according to crystalline form.

M. Haüy has made some interesting experiments on the means of detecting iron in mineral or other bodies by MAGNETISM.* Its presence may be ascertained by the attraction of the substance, immediately after having been heated in the flame of a taper, on the magnetic needle; but, in order to render the effect more sensible, M. Haüy has taken advantage of the combined forces of the magnetism of the earth, and of a bar magnet acting simultaneously on the needle. By numerous experiments and calculations of the relative repulsive power of the bar magnet on the needle, at different angles with the magnetic meridian, he found that the time of applying a mineral or other substance supposed to contain iron to the needle, is when it is retained at right angles with the magnetic meridian; because an effect will be

* *Annales des Mines*, No. 3.

produced there by a force many times smaller than that necessary to produce a similar effect on a needle uninfluenced except by the earth's magnetism. He found that in this way effects were produced on the needle by bodies that in common circumstances appeared to have no action, as hæmatite; the carbonate, phosphate, chromate, and arseniate, of iron; ferriferous carbonate of lime, garnet, peridot, &c.; and he observes that this extension of character, by means of double magnetism, may be usefully employed in the description of ferriferous minerals.

The nature and properties of the different species of rays existing in SOLAR LIGHT continue to be investigated by philosophers with much assiduity. A very interesting paper, by Dr. Brewster, was read to the Royal Society in January last, *on the laws of polarization in regularly crystallized bodies;* but, as it does not particularly relate to chemical science, I pass it over for the consideration of those discoveries more intimately connected with it. Mr. Playfair appears to have witnessed, when at Rome, the hitherto-doubtful experiment of Morrichini on the communication of magnetism to a steel needle by the violet rays of the solar beam. The time required to produce the effect was 55 minutes. A particular account of this experiment having been inserted in the 232d Number of your Journal, renders any further notice of it in this place unnecessary. I may however observe, that those experiments have been repeated by M. Ridolfi with successful results.

M. Berard has been engaged in conducting a series of experiments on the properties of the different rays of solar light, to demonstrate the relative situation and intensity of the heating rays, the rays of light, and the chemical rays; and the analogy which exists between them in their general physical characters. His account of the arrangement of the three sets of rays in the prismatic spectrum does not require notice, as it agrees with the results of the experiments of Herschel, Dr. Wollaston, and other philosophers in this country; but the laws of the polarization of the rays of heat, and the chemical rays, are not so well known. Your limits will not permit me to follow M. Berard through the account of his experiments; I shall merely state the results of them. I must however previously observe, that, having substituted a prism of calcareous spar for the one of glass used in former experiments, he found that in each image formed by the prism the red extremity was hotter than the violet; and this induced him to suspect that the rays of heat underwent a double refraction in the manner of the rays of light; and also that the rays of heat were polarized nearly under the same angle. His next experiment seemed to prove that radiant heat reflected by a glass at about an angle of 35°, and, falling on another glass making the same angle with its surface, is reflected by this second plane when it is turned in two positions opposite to each other, and is not reflected in two other positions equally opposed, each being intermediate and equally distant from the two first. Radiant heat, therefore, like light, may be polarized. The angle at which radiant heat is most completely polarized has not been exactly ascertained; but it appears not to differ much from that of light. M. Berard then substituted for the glass reflectors two polished metallic surfaces, and found that, in every position of the second surface, the thermometer rose nearly in the same degree. The angles of incidence were varied without any greater effect being noticed towards the east than in the south; from which it may be concluded, that to heat, as with light, metallic surfaces cannot communicate the same singular properties as may be given by surfaces of glass. The chemical rays of solar light were next examined. Having noticed the properties of those rays in effecting chemical changes, M. Berard mentions an experiment he made, which consisted in receiving the chemical rays directed in the plane of the meridian on a glass surface at an angle of incidence of 35° 6′. The rays reflected by this first glass were received on a second at the same incidence. He found that, when this was turned towards the south, the muriate of silver exposed to the reflected invisible rays, was blackened in half an hour; whilst, if it were turned towards the west, it was not at all discoloured in ten hours. The chemical rays may therefore be polarized, like the rays of light, by surfaces of glass under a certain angle, which appeared to be nearly the same for both kinds of rays. It may therefore be also presumed, that the chemical rays will suffer double refraction in passing through certain diaphanous bodies. (*Memoires de Arcueil, tome* 3.)

The doctrine of Sir Humphry Davy respecting the nature of FLAME, I presume, must be generally known. This enlightened philosopher has been lately engaged in some further researches on this subject. Flame he considers to be aërial matter heated to a certain point, at which it becomes luminous: and it appears that different temperatures are necessary to maintain the combustion of different gaseous bodies. When they are cooled below that temperature, they become extinguished. It is from this reason that a covering of wire-gauze prevents a lamp from exploding the carburetted hydrogen gas of coal mines, even when it is mixed with such a proportion of common air as to be at the exploding point. The heat is so much diminished by the wire, that the aërial matter which passes through it is too cold to be capable of setting fire to the coal-gas. When a hot platinum wire of a small diameter is put into an exploding mixture, it continues luminous for some time, the combustion going on around the wire, evolving sufficient heat to maintain the luminous temperature of the wire, but insufficient to explode the gaseous mixture. This curious fact has been applied to the maintaining what has been called a lamp without flame.* A few coils of platina, about 1-100th of an inch diameter, are placed around the wick of a spirit-lamp; after burning a short time, the flame is to be blown out; the wire immediately becomes luminous, and continues so until all the spirit is consumed.

The doctrine of the simple nature of CHLORINE continues to meet with opponents, some of whom are philosophers, ardent for the acquisition, and zealous in the pursuit, of knowledge. In the thirty-fifth volume of your Journal there is an account of the state of the question at that time, and the facts on which the diversity of opinions was founded; I have therefore only to state of what has been advanced respecting it since the period above alluded to. Dr. Ure, of Glasgow, a short time since, completed an elaborate series of experiments, which he considers decisive. He first passed the vapour of dry muriate of ammonia through thin laminæ of the pure metals—silver, copper, and iron, ignited in tubes of green glass out of the contact of the air; and found in each case the metal converted into a muriate, whilst a portion of water, nearly equal to one-sixth the weight of the dry salt, made its appearance. He found it impossible to obtain water by merely heating, or passing over heated charcoal or quartz, the salt resulting from the union of the acid and ammoniacal gases, when they had been previously dried. A desire having been expressed by some members of the Royal Society, that Dr. Ure would repeat his experiments with the salt resulting from gaseous combination, he did so, and with similar results. By transmitting dry muriatic gas over the ignited metals, Dr. Ure also obtained water, corresponding in quantity to the metallic muriate formed. He infers from his experiments, that muriatic acid gas is composed of a volume of dry acid combined with a volume of water, like the vapours of nitric and sulphuric acids, and that chlorine is oxygenated muriatic acid. Dr. Murray has also been engaged in the same enquiry. He repeated the abovementioned experiments of Dr. Ure with similar results; he also repeated an experiment made some years since, of obtaining

---

* Annals of Philosophy, vol. 11.

water from muriate of ammonia by heat,—employing an apparatus on the principle of Dr. Wollaston's Cryophorus, and with a successful result. He then submitted muriatic acid gas to various experiments. Iron-filings, perfectly dry and clean, were put into a glass tube surrounded with sand, and placed across a furnace, so as to be raised to a red heat: muriatic gas, extricated from a mixture of supersulphate of potash and muriate of soda, and conveyed through a tube containing dry muriate of lime adapted to the other, was transmitted over the ignited iron. Moisture immediately collected in the tube beyond the ignited space, and hydrogen gas was disengaged. In another experiment, the gas was previously kept in contact with muriate of lime for several hours, and was then passed over the ignited metal with a similar result.—Unless there be some fallacy in these experiments, it is obvious the results are incompatible with the doctrine of chlorine being a simple substance; for, according to that, muriatic acid gas is the pure acid, free from water. There is a difficulty also in explaining them on the principles of the opposite doctrine; for, according to that, the ignited metal should decompose the water, combining with its oxygen; and the products should be a dry muriate, or chloride, and *hydrogen gas*. Dr. Murray offered an explanation of this, by supposing that it might arise from the formation of a super-muriate, the quantity of water combined with the quantity of acid which forms a neutral muriate being sufficient for the oxidation of the metal; so that, if an additional portion of acid entered into the combination, the water of this might be liberated. It was accordingly found that the products in all these cases were acid. Dr. Murray thinks that the above results prove the fallacy of the doctrine of the simple nature of chlorine. The old opinion respecting it he considers might be held established. He has however offered another view of the subject, which is certainly very ingenious, and more conformable to the present state of chemical theory. It is now well ascertained, that oxygen cannot be regarded as exclusively the principle which communicates acidity; the same property is in several instances possessed by hydrogen. He observes, that, because water is obtained from muriatic gas, it does not necessarily follow that it has pre-existed in the state of water. It is equally possible that the elements of water only may have existed in the gas. On this view, oxymuriatic acid will be a compound of a radical, at present unknown, with oxygen; and muriatic acid a compound of the same radical with oxygen and hydrogen. The same view he extends to other acids which have been supposed to contain combined water.

Sulphurous acid is considered a compound of sulphur and oxygen; sulphuric acid, of sulphur, oxygen, and hydrogen; and nitric acid, of nitrogen, oxygen, and hydrogen. With each of these radicals, oxygen and hydrogen communicate acidity; their combined action appears to do so in a still greater degree. This explains the apparent difficulty in the old doctrine respecting oxymuriatic acid—that it is a weaker acid than the muriatic, though, as then considered, it had received an additional portion of oxygen. This opinion he thinks equally applicable to iodine; and that it accords better with the nature of the compounds of inflammable bodies with chlorine, than either of the other doctrines. Dr. Murray extended this view to the constitution of the alkalies, which we shall notice when we take those substances into consideration. Sir H. Davy has made a series of experiments on the principle of those above-mentioned of Dr. Ure, which shew that the water obtained was an accidental product, generated by the hydrogen of the muriatic acid gas meeting with oxygen, which was derived from sources not suspected by Dr. Ure. When tubes of flint-glass were employed for the transmission of the gas, the oxide of lead and the alkali in the glass furnished the oxygen; and a portion of it was also furnished by the atmospheric air existing in the apparatus. Chlorine, freed from vapour by muriate of lime, yields no water when acted on by metals; and the quantity of water furnished by muriatic acid was always found to be diminished in proportion to the care taken to remove the above-mentioned sources of oxygen.

In support of the doctrine of chlorine, which, according to strict rules of philosophising, must, in the present state of our knowledge respecting it, be considered as true, we have to state, that it has at length been adopted by Berthollet.

Dr. Ure has discovered that the brown liquid which drains from the salt the soap-makers obtain from their waste leys, by evaporation, for the soda manufacturers, is rich in IODINE. To obtain it, this liquid was heated to 230° Fahr. poured into a stone-ware bason, filling it one-half, and was then saturated with one-eighth by measure of sulphuric acid, previously diluted with its own bulk of water.—On cooling, a large quantity of crystals adhere to the sides and bottom of the vessel, chiefly sulphate of soda, a little sulphate of potash, a few oblong rhomboidal plates of hydriodate of soda, and precipitate of sulphur intermixed with them.

The cold liquid was filtered through a woollen cloth; to every twelve ounces were added 1000 grains of powdered black oxide of manganese: this mixture was put in a glass globe or mattrass, with another inverted over the mouth, a wide collar of wood being previously put over its neck to prevent the heat of the charcoal from affecting the upper vessel. The iodine, on the application of the heat, sublimes in great abundance; as the receiver grows hot, others are successively substituted: thus procured, it is washed out with water, drained on glass plates, and dried. From this quantity, 200 grains may be obtained. This may be purified by sublimation from dry quicklime.

M. Houton Labillardiere has observed, that hydriodic acid gas is capable of uniting with its own volume of proto-phosphuretted hydrogen gas. The two gases condense each other, and form cubic crystals of a white colour, which volatilized by a gentle heat. This compound is decomposed by exposure to the air, by water, by alcohol, and by most of the salifiable bases, the proto-phosphuretted hydrogen being evolved. Common phosphuretted hydrogen (the gas composed of an atom of hydrogen and an atom of phosphorus) likewise combines with hydriodic acid gas. One volume of this gas unites with two volumes of the acid gas. When this combination is decomposed, proto-phosphuretted hydrogen gas is evolved, and phosphurus is precipitated.

M. Bauhof, of Aarau, has published an account of some interesting experiments he has made on the action of alcohol on OXALIC ACID. He dissolved one part of oxalic acid in eight parts of alcohol, put the mixture into a retort, and distilled off the alcohol. This process was repeated five or six times, till at last the whole oxalic acid disappeared, and was converted into an oily looking substance, obviously a compound of oxalic acid and alcohol. This substance possesses the following properties:—Its colour is brownish yellow: its smell resembles that of the *sweet oil of wine:* its taste is nauseous, bitterish, and somewhat metallic: it is heavier than water, and falls in it like drops of oil; but is partially soluble in it by agitation. When recently prepared, it changes the vegetable blues to red; but this appears to arise from its containing uncombined acid, which may be separated from it by agitation with carbonate of lime. It is soluble in alcohol. Mixed with water and distilled in a retort, it becomes decomposed, and acidulous water comes over, leaving behind an acrid liquor, which on cooling deposits crystals of oxalic acid. Caustic ammonia produces a white precipitate with it, which appears to be a compound of the oily matter with ammonia. It is destitute of taste and smell, and is not soluble in either hot or cold water. Heated, it is volatilized without undergoing decomposition. Muriatic and sulphuric acids dissolved it when assisted by heat. The solution is transparent. Alkalies produce no precipitate with it. When boiled with potash or soda, it is not decomposed, nor is any ammonia disengaged. Mixed with a solution of potash, and distilled in a retort, it furnished a liquid containing ammonia and alcohol; and, when the portion remaining in the retort is saturated with muriatic acid, and mixed with muriate of lime, a copious precipitate of oxalate of lime is produced.

Mr. Dalton has continued his investigation of the PHOSPHURETS,* and has found, as far as can be deduced from experiments hitherto made, that there is but one combination of phosphorus and hydrogen. Those varieties generally noticed have been a combination of phosphorus and hydrogen, with more or less of *free* hydrogen: the latter may easily be separated from it. This is conformable to the doctrine of definite proportions, or, as it is generally termed, the *atomic theory*; which appears to be daily assuming a more stable character.

Returning to Mr. Dalton's experiments, it appears that pure phosphuretted hydrogen may be mixed with safety in narrow tubes (three-tenths of an inch diameter) with pure oxygen; and in due time the mixture may be transferred into any sort of vessel without explosion, and kept many hours without any chemical action. One volume of phosphuretted hydrogen requires, as near as he has ascertained, two volumes of oxygen for its complete combustion. Phosphoric acid and water are formed. Pure phosphuretted hydrogen, by being electrified for one or two hours, expands nearly one-third of its original bulk; phosphorus is deposited, and the residual gas is hydrogen, mixed with more or less of phosphuretted hydrogen. Water freed from air absorbs fully one-half of its volume of this gas: one volume of it, mixed with from two to five volumes of pure nitrous gas, affords a most brilliant explosion by the electric spark. With nitrous oxide it explodes by the electric spark, but undergoes no change by simple mixture, for several hours at least.

Experiments with this gas appear likely to determine the controverted question respecting the constitution of phosphoric acid, as well as those concerning the quantities of azote and oxygen in the nitrous compounds.

A paper by Sir H. Davy, was read to the Royal Society in April, containing an account of a series of experiments on the combinations of phosphorus with oxygen and chlorine. I shall give a general account of that part of it which relates

---

* Annals of Philosophy, No. 61.

to the experiments of M. Dulong, deferring a particular consideration until it appears in the Transactions of the Society. The author first endeavoured to discover the composition of phosphoric acid. The best way of accomplishing this, he found, is to burn the vapour of phosphorus, as it issues from a small tube, in oxygen gas. By adopting this process, he determined its composition to be 100 phosphorus to 134.5 oxygen. This will be found to differ from the proportions given by other chemists: Lavoisier stated the proportion of oxygen to 100 parts of phosphorus to be 154; Dr. Thomson, 123.37; and, M. Dulong, 123. Sir H. Davy then examined the phosphorous acid, which is supposed to contain half as much oxygen as the phosphoric acid, which is 67.25: Thenard stated it at 110; Gay-Lussac, 76; and Dulong, 74.88. He then enters upon the consideration of the acid announced by M. Dulong, under the title of hypo-phosphoric acid, and stated to consist of phosphorus 100, and oxygen 37.44 parts. Sir H. Davy is disposed to admit the existence of this as a proper chemical compound, but he thinks the analysis given of it by M. Dulong is not correct. With regard to the phosphatic acid of the French chemist, (stated as phosphorus 100, oxygen 112.4 parts,) he does not think its existence substantiated. From a comparison of different experiments, made on various compounds of oxygen and phosphorus, Sir H. Davy assigns 45 as the equivalent number for phosphorus; and, proceeding upon the principle, that the oxygen and hydrogen in water exist in the proportion of 15 to 2, he gives the following proportions: in the hypo-phosphoric acid, 45 phosphorus to 15 oxygen; in phosphorous acid, 45 to 30; and, in phosphoric acid, 45 to 60.

Previously to the consideration of metallic substances, I shall give an account of the doctrine proposed by Dr. Murray, respecting the agency of oxygen and hydrogen in communicating alkalinity: he observes, that alkalinity is, as well as acidity, a result of the agency of oxygen; the fixed alkalies, the earths, and metallic oxides, (all of which contain oxygen as a common element,) form a series in which there is no well defined line of separation. Ammonia stands insulated; it contains no oxygen, yet its alkaline properties are energetic; a circumstance which led him to believe that oxygen must exist in one or other of its constituent principles. He thinks, however, that it may be explained in another way: as hydrogen, like oxygen, communicates acidity, so it may, like oxygen, give rise to alkalinity. Ammonia, therefore, will be a compound, of which nitrogen is the base, deriving its alkaline quality from hydrogen; and hence stands in the same relation to the other alkalies that sulphuretted hydrogen does to the acids. The fixed alkalies—barytes, strontites, and lime, have been supposed to contain combined water, essential to them in their insulated form. It is probable, that the elements of water rather exist in direct combination with their metallic base: that potash, for instance, is a compound of potassium, oxygen, and hydrogen; and thus the entire class will exhibit the same relations as the class of acids. When an acid and an alkali unite, the hydrogen of both is expended in forming water: the neutral salts, according to these views, will, therefore, either be sur-compounds of two binary compounds,—one of the radical of the acid, the other of the radical of the alkali with oxygen; or they are ternary compounds of the two radicals with oxygen. The latter he considers the more probable opinion.

M. Fischer, of Schaffouse, has reduced manganese to the metallic state in considerable quantity, and gives the following description of its properties: the fracture is neither conchoidal nor crystallised, but irregular, and very similar to that of the sulphuret of iron, commonly called marcasite. The metal is of a white colour; it is harder than tempered steel, and cuts glass almost as well as the diamond; it is capable of scratching rock-crystal; it takes a very fine polish, but which is not permanent, in consequence of its affinity for oxygen; placed in water for twenty-four hours, it becomes covered with a brown oxide; it acts sensibly on the magnetic needle, perhaps in consequence of its containing a little iron: the specific gravity is 7.467. Dr. Marshall Hall, in a paper read to the Royal Society, mentions some experiments he had made on the action of water and oxygen on IRON, which seem to prove that pure water, below 212°, has no action on iron; but, if the water contains air, oxidation takes place from the decomposition of the air: he also shows, that nitrogen alone is evolved, and no hydrogen; and that, when water is deprived of atmospheric air or oxygen, iron retains a clear and bright surface, though exposed many months to its action.

It has been ascertained, by M. Chevreul, that the per-oxide of URANIUM is soluble in the alkaline sub-carbonates, and forms with that of potash a regularly crystallised salt: no carbonic acid is disengaged; the solution is of a fine yellow colour, similar to that of the chromate of potash.*

An enormous mass of native COPPER has been found in North America, by M. Francis le Baron, of the United

* Journal of Science and the Arts, No. 9.

States: it was discovered in the bed of the river Onatanagan, and measures twelve feet round at one end, and fourteen feet at the other. It is supposed to have been detached from some distant source, and brought by the force of the water to the situation in which it was found. The pieces that have been taken from it appear to be very pure; they are extremely ductile, malleable, and capable of being highly polished.* Mr. Phillips has been engaged in a series of experiments on the green and blue carbonates of copper, the results of which are interesting, particularly as applicable to the theory of definite proportions. Klaproth and Vauquelin had stated the green carbonate to be constituted of

| | Klaproth. | | Vanquelin. |
|---|---|---|---|
| Copper . . . . | 58. | — | 56.10 |
| Oxygen . . . . | 12.5 | — | 14. |
| Carbonic acid . . . | 18. | — | 21.25 |
| Water . . . . | 11.5 | — | 8.65 |
| | 100.0 | | 100.00 |

The statement of Vauquelin does not much vary from the results of the experiments of Mr. Phillips, which were

| | |
|---|---|
| Per-oxyd of copper . . | 72.2 |
| Carbonic acid . . . . | 18.5 |
| Water . . . . . . | 9.3 |
| | 100.0 |

Supposing it to be composed of one volume of oxyd, acid, and water, 100 parts would contain, according to the numbers determined on to represent those volumes,

| | |
|---|---|
| Per-oxyd of copper . . | 72.01 |
| Carbonic acid . . . | 19.82 |
| Water . . . . . . | 8.17 |
| | 100.00 |

This will be found to vary but little from the result of the experiments of Mr. Phillips. The analysis of the blue carbonate would lead us to fix the proportions of that as three volumes of per-oxyd of copper, four of carbonic acid, and two of water. He also examined the preparation of copper used by painters, called *verditer*, with the view of forming

* Journal of Science and the Arts, No. 9.

it by synthesis. The result was the same as that of the blue carbonate before mentioned; but he could not succeed in his attempt to produce it. Pelletier (*Ann. de Chimie, tom.* 13,) stated it to contain a portion of lime; but Mr. Phillips could not detect this substance in it.*

M. Lampadius states, that hydrogen-gas dissolves copper when it is passed over this metal in fine powder, at a white heat; the gas then burns with a green flame, and forms, during its combustion, an oxide of copper.

A new mode of preparing the SUBMURIATE OF MERCURY is recommended in the Journal of Science, which is as follows:—Prepare an oxy-sulphate of mercury by boiling twenty-five pounds of mercury with thirty-five pounds of sulphuric acid to dryness; triturate thirty-one pounds of this dry salt with twenty pounds four ounces of mercury, until the globules disappear, and then add seventeen pounds of common salt: the whole to be thoroughly mixed and sublimed in earthen vessels. Between forty-six and forty-eight pounds of calomel are thus produced: it is to be washed and levigated in the usual way.

M. Vauquelin observed, on dissolving a sulphuret of soda in water, which he had prepared in a PLATINUM crucible, a residuary black matter in the form of needles. When this was heated in the open air, it gave out the odour of sulphurous acid and left platinum behind; showing it to be a sulphuret of platinum which had been formed by the action of the sulphuret of soda on the crucible. He succeeded in forming the same substance by heating together, in a platinum crucible, equal weights of ammonia-muriate of platinum, sulphur, and carbonate of soda: sulphuret of platinum thus obtained was in the form of black brilliant needles. Heated in the open air, it loses from 15 to 16½ per cent. of sulphur: in close vessels, it undergoes no further change than a kind of fusion; it is not acted on by the simple acids. When a stream of sulphuretted hydrogen-gas is passed through a solution of platinum in an acid, a black powder is precipitated, which, when heated, gives out a portion of water and sulphurous acid, and then becomes a sulphuret similar to that prepared in the dry way.

When a solution of platinum in nitro-muriatic acid is exposed to heat, a submuriate of platina is formed, which is insoluble in water; it may be decomposed by potash and soda, leaving a black oxide, containing about 15 or 16 per cent. of oxygen.

When common muriate of platinum is exposed to a heat

* Journal of Science and the Arts.

sufficient to drive off a portion of its acid, chlorine is disengaged, and the salt acquires a fawn-brown colour, and loses its taste and solubility; and, when 100 parts of it are exposed to a strong heat, they leave 72.5 parts of metallic platinum. Two opinions may be entertained respecting the nature of this brown residue. It may be a chlorine of platinum, on which supposition it would be a compound of

| | | | |
|---|---|---|---|
| Chlorine . . . | 27.5 | — | 4.5 |
| Platinum . . . | 72.5 | — | 11.863 |
| | 100.0 | | |

Or, it may be a compound of muriatic acid and oxide of platinum. This is the opinion entertained by Vauquelin. If we suppose the oxide, as seems to follow from Vauquelin's experiments, to be a compound of

| | |
|---|---|
| Platinum . . . . | 84 |
| Oxygen . . . . | 16 |
| | 100 |

then the oxide of platinum in this salt must amount to 86.3, and of course it must be a compound of

| | | | | | |
|---|---|---|---|---|---|
| Muriatic acid . . | 13.7 | — | 4.625 | — | 9.250 |
| Oxide of platinum . | 86.3 | — | 29.134 | — | 14.567 |
| | 100.0 | | | | |

This last supposition would make the weight of an atom of platinum 12.567, if we consider the oxide to contain two atoms of oxygen.

When a solution of muriate of platinum, as neutral as possible, is mixed with the requisite proportion of common salt, a triple salt is formed, which crystallizes with facility in fine crystals of a beautiful orange-red. If, instead of common salt, caustic soda be added in such a proportion as not to be in excess, the liquid becomes of a dark-brown colour, but no precipitate takes place. When this liquid is allowed to evaporate spontaneously, yellowish-brown crystals are obtained, forming plates like mica, among which some may be perceived of a pearl-grey colour, and very brilliant. These crystals are perfectly neutral.

When sulphuric acid is mixed with muriate of platinum, if the mixture be boiled for a sufficient length of time, and a proper quantity of sulphuric acid be employed, the whole of the muriatic acid is driven off, and a sulphate of platinum formed. This sulphate, when concentrated, appears black; but it becomes green when diluted with water. It is deliquescent, and does not seem capable of crystallizing. When mixed with sulphate of potash, and evaporated, a green flocculent precipitate is formed, and the liquid becomes almost colourless. This precipitate is a triple compound of sulphate of platinum and sulphate of potash.*

Mr. Hume has continued his investigation of the means of detecting the presence of ARSENIC when in small proportions, or mixed with various extraneous matters, as the contents of the human stomach. The results of his very important and accurate experiments were communicated for this Journal, and will be found in the late Numbers, with observations on them by other correspondents.

Brugnatelli has proposed a test which appears to be a very delicate one:—To a mixture of fresh wheat-starch and water, add a sufficient quantity of iodine to give the liquid a lively blue colour; a solution of white arsenic added to this, turns it to a reddish colour; the same change is also effected by a solution of sublimate of mercury. When the change has been produced by arsenic, the blue colour will be restored by the addition of a few drops of sulphuric acid; but this will not happen when sublimate has been employed.†

It is stated in the Journal of Science, ninth number, that CHROMIC OXIDE heated with an alkali becomes chromic acid, and chromic acid heated with an acid becomes chromic oxide; the oxide in solution is green, and the acid yellow, and the change of state and colour may be produced successively at pleasure. I understand that Mr. Brande has been engaged in a series of experiments with this interesting substance, and has made some important discoveries respecting it; from these we may expect an explanation of the above phenomena.

A new mineral, called PARGASITE, has been sent to this country from Finland. It was found some years ago at the village of Ersby, near Abo. It is of a green colour, and is transparent. Its crystals are of various sizes, from an inch downwards. Its form is an octohedron, with a rhomboidal base. It has three cleavages, is harder than fluor spar, but is scratched by quartz; it also scratches glass. Specific gravity is 3.11. It melts before the blow-pipe into a mass of a pearly white lustre.

---

* *Ann. de Chimie.* † Ibid.

The following are given as the proportions of its constituents:

| | |
|---|---|
| Silex | 42.01 |
| Magnesia | 18.27 |
| Lime | 14.28 |
| Alumina | 14.08 |
| Oxide of iron | 3.52 |
| ——— manganese | 1.02 |
| *Oxide of a metal not investigated* | .33 |
| Fluoric acid and water | 3.90 |
| Loss | 2.59 |
| | 100.00 |

The term LEELITE has been given by Dr. Clarke to a red silicious stone, lately brought to this country from Gryphylta, in Sweden; described by some of the Swedish chemists as a pure hydrate of silica, of the same nature as opal. It is of a red colour, possessing no more lustre and transparency than horn. Its fracture resembles that of flint, which it also equals in hardness: specific gravity is 2.71. It was found by analysis to consist of

| | |
|---|---|
| Silica | 75.00 |
| Alumina | 22.00 |
| Manganese | 2.50 |
| Water | .50 |
| | 100.00 |

Another new mineral has been received from the same country, which has been called PETALITE. Dr. Clarke,* to whom it was sent, describes it as having nearly the appearance of *quartz,* which it also resembles in specific gravity. It admits of a two-fold cleavage parallel to the sides of a rhomboidal prism, two of which parallel to each other are splendent, and the other two dull. Its angles are equal to 100° and 80°, and its form that of a four-sided prism with a rhomboidal base. Its colour is white with a slight pink hue; it is hard enough to cut glass, although it may be rased with a knife. It furnished by analysis, in round numbers, silicia 80, alumina 15, manganese 3, and oxide of an unknown metal 2. The latter has been found by M. Arfvedson, an ingenious young Swedish chemist, to be a metal hitherto unnoticed, the oxide of which is an alkali, possessing many peculiar properties. Its combinations with acids are

* Annals of Philosophy, No. 63.

very fusible; the sulphate and muriate liquify before they arrive at a red heat; the carbonate just when it begins to become red. The muriate is more deliquescent than that of lime. Its nitrate forms rhomboidal crystals, which attract the moisture of the atmosphere with avidity. Its carbonate is with difficulty soluble in water. It has a greater capacity of saturating acids than any of the other alkalies. This has been called *Lithion* by the Swedish chemists, from its having been discovered in the mineral kingdom. According to the general construction of our chemical nomenclature, I should term it *Lithina*.* This substance has been procured by Sir H. Davy, who found its properties agree with those stated by M. Arfvedson. He has also reduced it to the metallic state, in which it bears a strong resemblance to sodium. M. Vauquelin has also procured it; he found it to contain 43.5 of oxygen; a greater proportion than any of the other alkalies.

The same number of the *Annales de Chimie* contains an account of *an acidifiable metallic substance*, discovered by Berzelius in the pyrites from the mine of Fahlun, which he has called *Selenium*. He first obtained it from the red precipitate formed during the burning of the pyrites for the manufacture of sulphur. He at first supposed it to be a mixture of tellurium with sulphur; but, on more minute examination, it proved a combination of the latter with selenium. When viewed in a mass, it is of a grey colour, with a brilliant metallic lustre: its fracture is vitreous, like that of sulphur, and displays a reddish tinge. The specific gravity 4.6. In hardness and friability it is nearly similar to sulphur. It may be reduced, by trituration, into a red powder, interspersed with particles having a metallic lustre. It becomes soft at the temperature of boiling water, and melts at a higher degree of heat. During cooling, it preserves for some time a degree of fluidity, like sulphur, or Spanish wax; so that it may be moulded between the fingers, and drawn into threads of a brilliant metallic lustre, which, by transmitted light, are of a deep-red colour. In a still higher degree of heat it sublimes in opaque metallic globules, and the globe of the retort is filled with a yellow gas. Distilled in a large necked retort it sublimes in flowers of a beautiful vermilion colour, which however are not oxidized, for the metal may be obtained in its original state by simple fusion. Sublimed in the air, without the contact of fire, it evaporates in a red smoke, without any particular odour; but, if a candle be applied, or it be exposed to the blow-

* *Annales de Chimie, Fevrier.*

pipe, it burns with an azure-blue-coloured flame, and exhales so powerful a smell of radish (*raifort, raphanus sativus*), that one-fiftieth of a grain, treated in this manner, is sufficient to fill a large room with it. It combines with other metals, and generally produces a reddish flame. Selenuret of potash forms a metallic button of a greyish-blue colour, which dissolves rapidly in water without evolving any gas, and produces a fluid of a reddish colour, having the taste of hydro-sulphuret of potash. The acids disengage from it a gas, which, when diluted, may be mistaken for sulphuretted hydrogen gas; but if taken into the nostrils undiluted, even in a very small quantity, it produces violent inflammation. The hydro-selenuret of potash dissolved in water, becomes covered with a pellicle that is at first of a vermilion colour; but as it thickens it changes to grey. On the addition of muriatic acid, the solution becomes turbid, and deposits a red powder, proving that it contains an excess of the new substance in solution; the same thing takes place with the hydro-sulphuretted sulphurets.

Selenium combines with the fixed alkalies, both in the humid way and by fusion. These combinations are of a vermilion colour. It also dissolves in the fat oils: these solutions are red, but have no hepatic odour, like those of sulphur. It dissolves in nitric acid by the assistance of heat; the solution evaporated forms a salt, which sublimes crystallized in needles, which are soluble both in water and alcohol, of a pure acid taste, reddening the tincture of turnsole, and forming with alkalies specific salts. The alkaline selenates are crystallized with difficulty, and attract the humidity of the air. The selenate of ammonia, exposed to heat, disengages a small proportion of ammonia; the selenic acid is afterwards sublimed; the greatest part of the ammonia is decomposed, disengaging water and azotic gas; the selenium is left behind in a state of fusion, which may be sublimed by a higher degree of heat. The selenate of barytes is soluble in water: it crystallizes in needles, the extremities of which become covered with a pencillated group of others of a smaller size, the interstices of which are gradually filled up, and, in this manner, a crystal of a globular shape is formed. If muriatic acid be poured into a solution of a selenate, and a piece of zinc be afterwards immersed in it, the selenium is precipitated in a metallic state; the zinc at first appears covered with a pellicle of copper; afterwards the selenium is deposed in vermilion flakes. With sulphuric acid the precipitate is made with more difficulty, assumes a grey colour, and contains sulphuret of selenium. Sulphuretted hydrogen-gas, passed through a solution of selenic acid, gives a precipitate of an orange colour, becoming red by exsiccation, which sublimes in a transparent orange-coloured mass.

The pyrites of Fahlun, employed for the extraction of sulphur, are combined with galena; and it is probable that this contains selenium under the form of selenuret of lead. The quantity of selenium contained in these minerals is very small: five hundred pounds burned at the manufactory furnished only sixty grains of selenium.

Professor Berzelius has made some experiments with this very singular and interesting body. He states* that the combinations of selenium with the alkalies and the alkaline hydro-selenurets (hydro-seleniates according to the nomenclature of Gay-Lussac), elucidate many points of theory. Selenium may be combined with potash without a seleniate and a seleniated hydro-selenuret being produced from it when dissolved in water; yet this combination possesses similar properties to the alkaline sulphurets; from which it seems to follow, that the opinion of the dry sulphuret of potash being a mixture of sulphate or hydro-sulphate of potash with the sulphuret of potassium, is erroneous. The hydro-selenurets have the same taste and general properties with the hydro-sulphurets and the hydro-tellurets. It appears, therefore, that the hepatic taste, the spontaneous decomposition from the contact of the air, &c. characterize the compounds of those acids into which hydrogen enters in place of oxygen. It is a non-conductor of electricity and caloric. M. Berzelius states, that he has found a mineral which contains one-fourth its weight of this substance.

M. Stromeyer has discovered a new metal in the ores of zinc, which he has called CADMIUM. It has the appearance of tin, and in its chemical combinations resembles zinc, which precipitates it from its solutions, in the metallic state.

The only information I have to communicate on the subject of MINERAL WATERS is an account of the waters of Caldas de Rainha, in the province of Estremadura, fourteen leagues north of Lisbon. The temperature of the water when fresh drawn is 85° in a tumbler, but a thermometer, plunged immediately into the source, indicated a temperature of from 93° to 94°. The smell and taste are similar to that of Harrowgate water, but the water is less sparkling. The colour of a bright silver spoon was sensibly changed when held in the vapour, and the surface of a knife was readily acted upon when immersed in the water. It is entirely deprived of its smell by boiling. The spring yields upwards

* Annals of Philosophy, No. 66.

of 45 cubic feet per minute. Dr. Withering published an account of this water;* but his apparatus was very imperfect. His analysis of a gallon of the water is as follows.

| | |
|---|---|
| Fixed air . . . | $\frac{1}{4}$ oz. measure. |
| Hepatic air . . . | $6\frac{1}{4}$ |
| Calx aërata . . . | $3\frac{1}{2}$ grains |
| Ferrum hepaticum . . | $2\frac{1}{2}$ |
| Argil earth . . . | $1\frac{1}{4}$ |
| Siliceous earth . . | $\frac{3}{4}$ |
| Magnesia salt . . . | 64 |
| Selenetic salt . . . | 44 |
| Common salt . . . | 148 |
| | 264 grains. |

Some of the water brought to England was found to be of specific gravity 1005.8: 16 ounces evaporated gave 24 grains of dry salt, which were

| | |
|---|---|
| Muriate of soda . . | 12.2 |
| Sulphate of soda . . | 5.5 |
| Sulphate of lime . . | 4.1 |
| Sulphate of magnesia . | 1.7 |
| | 23.5† |

### Vegetable Chemistry.

There is but little to communicate on this subject. Some of the French chemists have lately analysed the different species of lichen, the gum of the olive tree, garlic, pimento, pyrethrum, the different species of corn, and some of the usual articles of diet; but I will not occupy the pages of this Journal with matters so uninteresting to the generality of readers.

Dr. Branchi, Professor of Chemistry in the University of Pisa, has discovered a volatile concrete oil in the nut-galls of the oak. It is white, of the same odour as pounded galls, and of a consistence nearly similar to butter of cocoa. It has a bitter caustic taste, which it imparts to water. Alcohol readily dissolves it. The solution is rendered turbid by the addition of a small quantity of water, but becomes transparent by a more copious dilution. It combines with the

* *Analyse da Agoa da Caldas da Rainha,* p. 64, 4to. 1795.

† Journal of Science and the Arts, No. 9.

fixed and volatile oils. It renders paper unctuous and diaphanous, which disappears when exposed to the atmosphere. It reddens turnsole paper. A solution of it is neither rendered violet nor black by sulphate of iron. It was found in Italian, Aleppo, and Istria galls, but most copiously in the latter. Six ounces furnished somewhat less than one grain.*

MM. Orfila and Majendie have completed their laborious investigation of the nature and properties of MORPHINE. The readers of this Journal will recollect that M. Sertuerner had discovered in opium a peculiar alkaline substance, which he termed *morphine*, this, by its union with an acid called *meconic*, contained in the same substance, appears to be the really narcotic part of opium. It appears by their experiments, that it exerted but little influence on dogs when given alone, owing to its slight solubility in water; but the same quantity, when combined with an acid, particularly the acetic, produced very violent effects. It was also tried when combined with olive oil; twelve grains exhibited in this way, destroyed a dog in eighteen hours. The following is an abstract of the conclusions M. Orfila drew from his experiments.

Morphine may be introduced into the stomach of a dog, to the extent of twelve grains as a dose, without producing any very sensible effect; while the same quantity of extract of opium usually causes death: but morphine dissolved in acetic acid, exerts on the animal economy a more powerful action than the same quantity of aqueous extract of opium. The extract of opium from which morphine has been separated, may be administered in a large dose without producing serious effects. Six grains of morphine, dissolved in oil of olives, appear to act more powerfully than twelve grains of the aqueous extract of opium. It exerts more violent action when injected into a vein than when taken into the stomach.

M. Majendie has employed the acetate and sulphate of morphine in several cases, with the most beneficial effects. It appears to act better, and occasion less unpleasant symptoms than opium. The dose of the acetate is from an eighth of a grain to half a grain: the sulphate is less powerful.

M. Majendie, in conjunction with M. Pelletier, has also subjected the different species of IPECACUANHA to chemical analysis. They found it to contain about 16 per cent. of a peculiar principle in which its emetic power consists; this

* Philosophical Magazine, vol. 50.

they have called *Emetine.* The following table shews the results of their analysis:

| | |
|---|---|
| Oils . . . | 2 |
| Emetine . . . | 16 |
| Wax . . . . . | 6 |
| Gum . . . . | 10 |
| Starch . . . | 42 |
| Woody fibre . . | 20 |
| Loss . . . . . | 4 |
| | 100 |

Emetine is, when dry, in the form of transparent scales, of a reddish brown colour. It has scarcely any smell. It is of a bitter, slightly acrid taste. Water dissolves it in great abundance, but no crystals can be obtained from the solution. It is dissolved readily in alcohol; but is insoluble in sulphuric ether. Diluted sulphuric acid has no action on it. It readily dissolves in the nitric, muriatic, phosphoric, and acetic acids. It is precipitated from alcohol and water by the gallic acid and the infusion of galls. It is also precipitated by acetate of lead, protonitrate of mercury, and corrosive sublimate. Half a grain of it excites violent vomiting, which is followed by sleep. Six grains given to a dog, occasioned the death of the animal in 30 hours: the mucous coat of the intestines and lungs were found in a state of violent inflammation.*

Some interesting experiments have been instituted by Dr. Gorham on the principle obtained from indigo, called INDIGOGENE.† Brugnatelli, who first discovered it, considered it as a metallic substance; and Dobereiner stated, that it was capable of amalgamating with quicksilver; but these opinions are incorrect. Its union with metallic substances is merely the result of cohesive attraction. Dr. Gorham's mode of preparing it, is to expose powdered indigo in a glass capsule to the heat of a lamp; a small quantity of water is first vaporized, after which there follows a bluish smoke of an offensive odour, the mass swells, and is apparently fused, and a purplish red vapour is observed to play over its surface; the lamp is to be withdrawn; indigogene condenses in crystals, and it may easily be detached from the mass beneath by a thin spatula. One hundred grains of the best indigo will yield from fifteen to eighteen grains; for nice experiments it requires a second

* *Annales de Chimie,* iv. 172.

† New England Journal.

sublimation, to free it from scoriæ. Thus procured, it appears an aggregate of small prismatic crystals, interlarded, of a purple colour, and slightly irridescent. The colour of a single crystal is a golden yellow; its specific gravity is about 2.6; when exposed to the temperature of 360° Fahr., it is volatilized, its vapour is dense, and it speedily crystallizes. At this degree of heat it is partly decomposed, a portion of it becoming semi-fluid, and finally concreting in the form of a black, spongy, and shining mass. It is insoluble in water, soluble in the acids, alcohol, sulphuric ether, oil of turpentine, and the fixed oils at a high temperature: dissolved in sulphuric acid, and the solution heated to the boiling point, it is decomposed and *carbon* is extricated. A mixture of it with cyanide (*prussiate*) of mercury, exposed to heat, gave out a strong odour of *hydrocyanic* acid; some minute globules of mercury passed into the receiver, and indigogene mixed with quicksilver remained behind. It is decomposed by chlorine, dry chlorine gas being mixed with its vapour, a dense yellow cloud immediately formed, the odour of hydrochloric acid became perceptible, and the receiver, in which the vapour was condensed, was found to contain drops of a yellow liquid, of an austere, disagreeable taste. Mixed with chlorate of potash, struck forcibly on an anvil, it explodes, and the mixture deflagrates brilliantly on exposure to a strong heat. The results of its combustion are carbonic acid, carbonic oxyd, and aqueous vapour; from which it may be concluded that its elements are carbon and hydrogen.

Its vapour is inflammable, burning with a dense red flame, exhaling a thick smoke. It decomposes the metallic oxyds when exposed to heat, in conjunction with them, and the salts which contain a large proportion of oxygen, by percussion. Protochloride of mercury (*calomel*) sublimed through indigogene, passed without any apparent reciprocal action; triturated with the perchloride of mercury (*corrosive sublimate*), and exposed to the heat of a spirit lamp, white vapours were given out, and the sides of the receiver became covered with a green film, which by the microscope was found to be crystals, in the form of flattened prisms. With a solution in alcohol, nitrate of silver and ammonia produced a white, and potash a deep yellow, precipitate. Water boiled on the portion left undissolved by the alcohol, and which still retained its colour, was not affected by the addition of potash, or ammonia. Thrown into a solution of potash, it remained unchanged; with sulphuric acid it formed a green solution, which changed to blue, on the addition of water. Exposed to heat, purple fumes, succeeded by a white vapour were produced, and the whole speedily dissipated. From the phenomena exhibited by this saline substance, he doubts whether it be a mixture of indigogene with the perchloride, but rather a triple compound of indigogene, chlorine, and metallic mercury? A very curious fact, with respect to the development of colours, is shewn by a solution of indigogene in sulphuric acid: this is of a fine green colour; but, on exposure to the air for a few hours, it becomes of a deep blue, and the same change is immediately produced by the addition of water. That this does not arise from chemical decomposition is evinced by combining them over mercury, when no gas is given out: nor does this change in colour take place, when it is exposed to the action of oxygen, hydrogen, nitrogen, or carbonic acid. It is known that cloths, dyed with indigo, are first green, but become blue after some days exposure to the atmosphere: this probably arises from the absorption of water, the effect being rather mechanical than chemical. When the sulphuric solution is largely diluted with water, and allowed to rest some hours, the indigogene is precipitated, the liquid becomes light blue, and is colourless when passed through paper. In the concentrated state, the solution is decomposed by potash, soda, ammonia, and lime-water, the indigogene being precipitated of a beautiful violet colour. Its solution in oil of turpentine is a fine green; that in sulphuric ether and alcohol, light blue; which perhaps arises from those fluids containing a portion of water. That in fixed oil is first light blue, but, as the heat is increased, it becomes greenish blue, then amethystine, and finally a deep and beautiful purplish red.

### Animal Chemistry.

M. Berard has subjected several animal substances to analysis by heating them in a glass tube previously mixed with peroxide of copper. The following is the result of his experiments.

| | Constituents by weight. | | | | |
|---|---|---|---|---|---|
| | Azote. | Carbon. | Oxygen. | Hydrogen. | TOTAL. |
| Urea ...... | 43.40 | 19.40 | 26.40 | 10.80 | 100 |
| Uric acid ... | 39.16 | 33.61 | 18.89 | 8.34 | 100 |
| Butter ..... | 00.00 | 66.34 | 14.02 | 19.64 | 100 |
| Fat........ | .... | 69.00 | 9.66 | 21.34 | 100 |
| Mutton suet | .... | 62.00 | 14.00 | 24.00 | 100 |
| Cholesterine | .... | 72.01 | 6.66 | 21.33 | 100 |
| Cetine .... | .... | 81.00 | 6.00 | 13.00 | 100 |
| Fish oil .... | .... | 79.65 | 6.00 | 14.13 | 100 |

M. Berard found urate of barytes to be composed of

| | |
|---|---|
| Uric acid . . | 61.64 |
| Barytes . . | 38.36 |
| | 100.00 |

Urate of potash—

| | |
|---|---|
| Uric acid . . | 70.11 |
| Potash . . . | 29.89 |
| | 100.00 |

Dr. Prout has subjected UREA, and LITHIC and URIC ACIDS to a careful analysis, a full account of which was given in this Journal in the review of the Transactions of the Medico-Chirurgical Society.

M. Berard has also made some experiments with the view of forming fat by an artificial combination of its elements. He mixed together one volume of carbonic acid, 10 of carburetted hydrogen, and 20 of hydrogen; and passed the mixture through a red hot porcelain tube. He obtained a substance in small white crystals, lighter than water, soluble in alcohol, and fusible by heat into a substance similar to fixed oil. Dobereiner has also made some experiments with the same view with the gas of coal; and it is stated, that he has obtained from it a substance very similar to fat. He also expects to obtain alcohol from the same substances.*

Berzelius has lately published in the *Annales de Chimie*, an interesting paper on the colouring matter of the BLOOD. He thinks the use of sulphuric acid in separating the colouring matter, as employed by Vauquelin, is quite unnecessary, and is by no means well adapted for the purpose. His method is to place a clot of blood upon blotting paper, in order to get rid of the serum as completely as possible; it is then put into water. The colouring matter becomes diffused in the water, while the fibrin remains; and by then evaporating the water, the colouring matter may be obtained in a separate state. No iron can be discovered in it while in this form, but when reduced to ashes it may be readily detected, in the proportion of one half per cent.

### MATERIA MEDICA.

I have to notice the introduction of some new articles into the list of Materia Medica, and the revival of the use of

* *Annales de Chimie*, July 1817.

others, that, although at a former period generally employed, had for some time past fallen into disuse.

The late numbers of this Journal contain much information respecting the medicinal qualities of prussic acid, the vapour of tar, green tea, James's powder, muriate of soda, and the preparations of gold.

The beneficial effects of DIGITALIS, as a local application in scrophulous ulcers, appear to be clearly evinced in an account published in a late periodical work (the Annals of Medicine), by Mr. Couch, of Polperro, in Cornwall. A strong decoction of the leaves was the form in which it was employed. The efficacy of digitalis in this respect, was very highly spoken of by some of the older English writers on Materia Medica; and it is now a popular remedy among the lower class of people in various parts of Ireland.

The ALISMA PLANTAGO (water plantain), is introduced to public notice as a remedy for hydrophobia, by Lewsheim, a Russian nobleman. The following is a translation of his account of this remedy, and directions for the use of it.

"Take a good-sized root, and two or three small ones; pound or reduce them into a fine powder, which spread on a piece of buttered bread and give it to the patient. Two doses, or three at most, are sufficient to eradicate the virulency of the poison, let it be ever so violent, even if the patient be already in the worst state, so as to be afraid of water. The efficacy of this root cures also animals bitten by mad dogs, and even mad dogs themselves. During the last twenty-five years, this remedy has not once failed; but has uniformly been a sure means of successfully restoring every person to their former health without any bad consequences afterwards; even those, who, from the violence of the poison, rushed upon people and bit them; which facts are particularly ascertained in the government of Toola.—The plant may be gathered during the whole of the summer, but it operates more efficaciously if gathered at the latter end of August; the roots of it, being taken up and washed clean from mud or other earthy matter, must be dried."

The efficacy of drinking the blood of the rabid animal, is also accredited in many parts of Russia, and cases in which it is said to have succeeded, both in preventing and removing *hydrophobia*, are related in the continental Medical Repertory, by Drs. Rittmeister and Stockman. Many superstitious and imposing ceremonies were, at the same time, employed, to which, (admitting the animals by whom the patients were bitten to have been rabid, and those cases which were called hydrophobia to have been such,) the efficacy of this remedy may most probably be attributed,

The numerous well authenticated instances we have of the occurrence of this disease without having been preceded by the causes usually assigned for it; and the remedies mentioned by Pliny, in the twenty-ninth book of his Natural History (which we cannot suppose were adduced without some foundation), will not permit us to doubt, but that it may be both induced and removed by the influence of the imagination.

---

