# Errata

**Published:** 1818-12

**Authors:** 


					Page 102, line 15, for atonic read atomic.

				

